# MRA-detected intracranial atherosclerotic disease in patients with TIA and minor stroke

**DOI:** 10.1007/s11845-022-03094-8

**Published:** 2022-07-15

**Authors:** Philip J. Dempsey, Mark C. Murphy, Michael Marnane, Sean Murphy, Eoin C. Kavanagh

**Affiliations:** 1 Department of Radiology, Mater Misericordiae University Hospital, Eccles Street, Dublin 7, Ireland; 2 Stroke Department, Dublin Neurovascular Institute, Mater Misericordiae University Hospital, Eccles Street, Dublin 7, Ireland

**Keywords:** Intracranial atherosclerotic disease, MRA, TIA, Stroke

## Abstract

**Objectives:**

Patients with TIA and minor stroke commonly undergo CT and CTA in the emergency department with subsequent MRI with MRA for further workup. The purpose of this study was to review outpatient MRIs for TIA/stroke patients to assess the additional benefit, if any, of the MRA sequence in the detection of intracranial atherosclerotic disease in patients for whom CTA had already been performed.

**Methods:**

The radiology reports of outpatient MRIs of the brain for TIA/minor stroke patients were retrospectively reviewed via the hospital PACS system. Following this, the imaging report from the patient’s initial presentation to the emergency department was reviewed. This index imaging and subsequent MRI were compared to assess the incidence of new vascular findings detected on the MRA sequence in patients for whom CTA had already been performed. Where new lesions had been identified at follow-up, the imaging was retroactively reviewed to assess if they were present on the index imaging.

**Results:**

Two hundred seven consecutive patients were reviewed. Significant (> 50%) intracranial atherosclerotic disease was present on MRA in 18 patients (8.7%). This was a new finding in 11 patients. Five had initial CTA where the atherosclerosis was not detected. All 5 of these cases were located in the posterior cerebral arteries. Incidental aneurysms were seen in 14 (6.7%); 12 were a new finding at time of MRI.

**Conclusion:**

The MRA sequence provides additional value by increasing the detection of clinically important intracranial atherosclerotic disease which may inform management in patients with minor stroke and TIA.

## Introduction

Advances in imaging have revolutionised the management of stroke and transient ischaemic attack (TIA). Patients presenting to the emergency department with symptoms of TIA undergo CT of the head to exclude intracranial haemorrhage and assess for early ischaemic changes. This is often accompanied by CT angiography (CTA) from the aortic arch to the vertex or Doppler ultrasound of the neck to identify a vascular culprit which will inform treatment decisions. MRI is an important tool in the diagnostic pathway of patients with suspected minor stroke or TIA, as it may not only confirm the diagnosis, but also point to an aetiology and guide therapy [1, 2]. When investigating minor stroke and TIA, MRI protocols commonly employ gradient echo, diffusion-weighted, fluid sensitive and MR angiography (MRA) sequences. Increasing the efficiency of diagnostic pathways for these patients is essential for prompt risk factor identification and appropriate management.

In this institution, we provide an early outpatient MRI pathway for TIA patients. The purpose of this study was to review the outpatient MRI of these rapid discharge TIA/stroke patients to assess if there was any additional benefit to the MRA sequence in the diagnosis of intracranial atherosclerotic disease (ICAD) in patients for whom CTA had already been performed.

## Materials and methods

### Study group

Patients who are seen in the emergency department with suspected TIA undergo initial imaging with non-contrast enhanced CT head to outrule intracranial haemorrhage. This may be accompanied by CT angiography from arch to vertex or carotid Doppler ultrasound. These patients are reviewed by the stroke service and may be discharged on the same day with early outpatient MRI. This clinical pathway is in place to facilitate rapid discharge with early outpatient clinical follow-up in appropriately selected patients.

### Data collection

A retrospective search of the hospital PACS was performed. All outpatient MRI brains performed between December 2018 and April 2020 were reviewed. The imaging referrals were then screened to ensure only early discharge minor stroke and TIA pathway patients were included. Once these patients had been identified, the outpatient MRI radiology report was reviewed. Specifically, findings pertaining to the MRA sequence were assessed. Time of flight intracranial (TOF) MRA was the technique employed for stroke protocol MRI. Following this, the report of the imaging from their initial presentation to the emergency department was reviewed. The findings at index CT and subsequent MRI were compared to assess if additional vascular findings had been identified on the later imaging by the TOF MRA sequence that had not been noted on the initial CT. Where differences existed between the findings reported (for example, an intracranial stenosis identified on the follow-up MRI but not mentioned in the initial CTA report), the studies were reviewed by three readers, a consultant radiologist with neuroradiology expertise and two radiology registrars in training. Discrepancies were resolved by consensus. The primary outcome of interest was the incidence of new ICAD detected on the TOF MRA sequence.

## Results

Over a period of 17 months, 207 consecutive patients were reviewed. All had an initial non-contrast CT brain followed by an MRI. One hundred of these patients had a CTA performed during their initial assessment. The average time from the initial presentation to outpatient MRI was 6 weeks. The earliest outpatient MRI took place 2 days post-discharge and the latest 35 weeks. The findings on MRA are outlined in Table 1.

**Table 1:** Findings on follow-up MRA

Findings
18 Significant intracranial atherosclerotic stenosis (ICAS) (8.6%)
• 11 > 50% stenosis
• 9 > 70% stenosis
Further 21 non-stenotic intracranial atherosclerotic disease (ICAD) in cavernous portions of the carotid arteries
14 incidental aneurysms (6.7%)
Size range: 2–7 mm

**Fig. 1:**
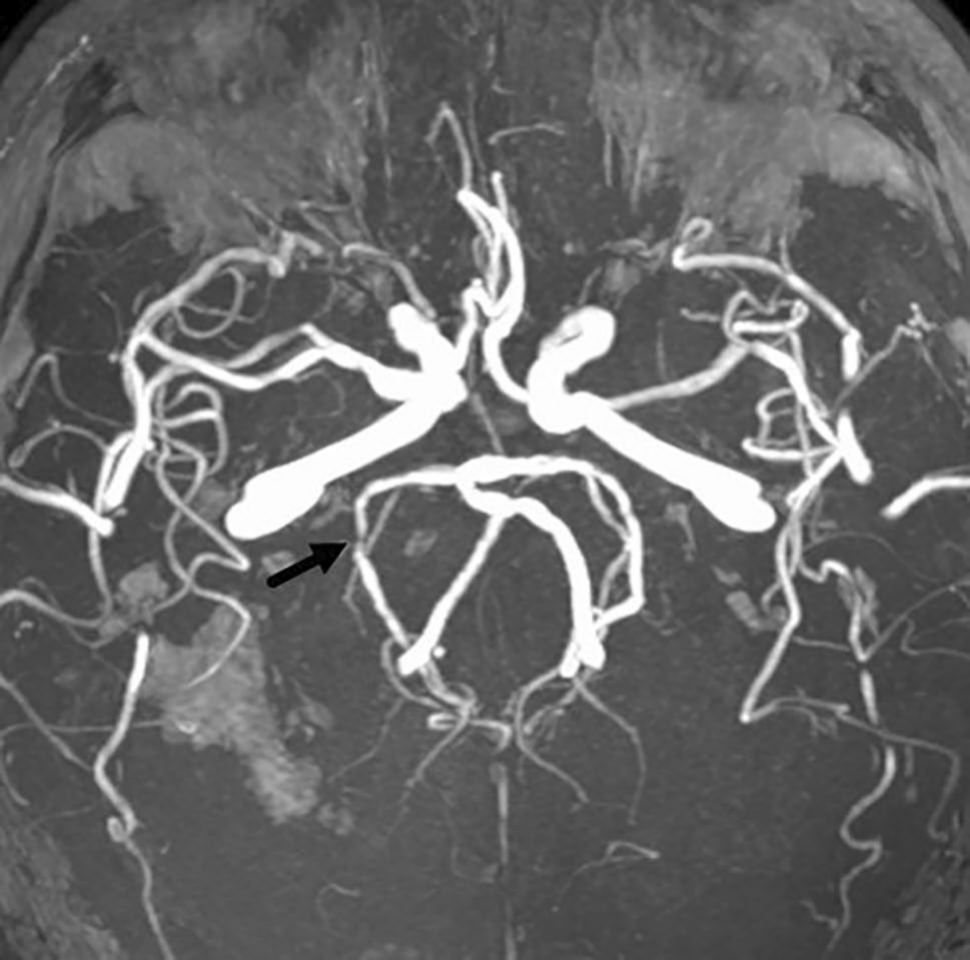
MIP TOF demonstrating right PCA stenosis

**Fig. 2:**
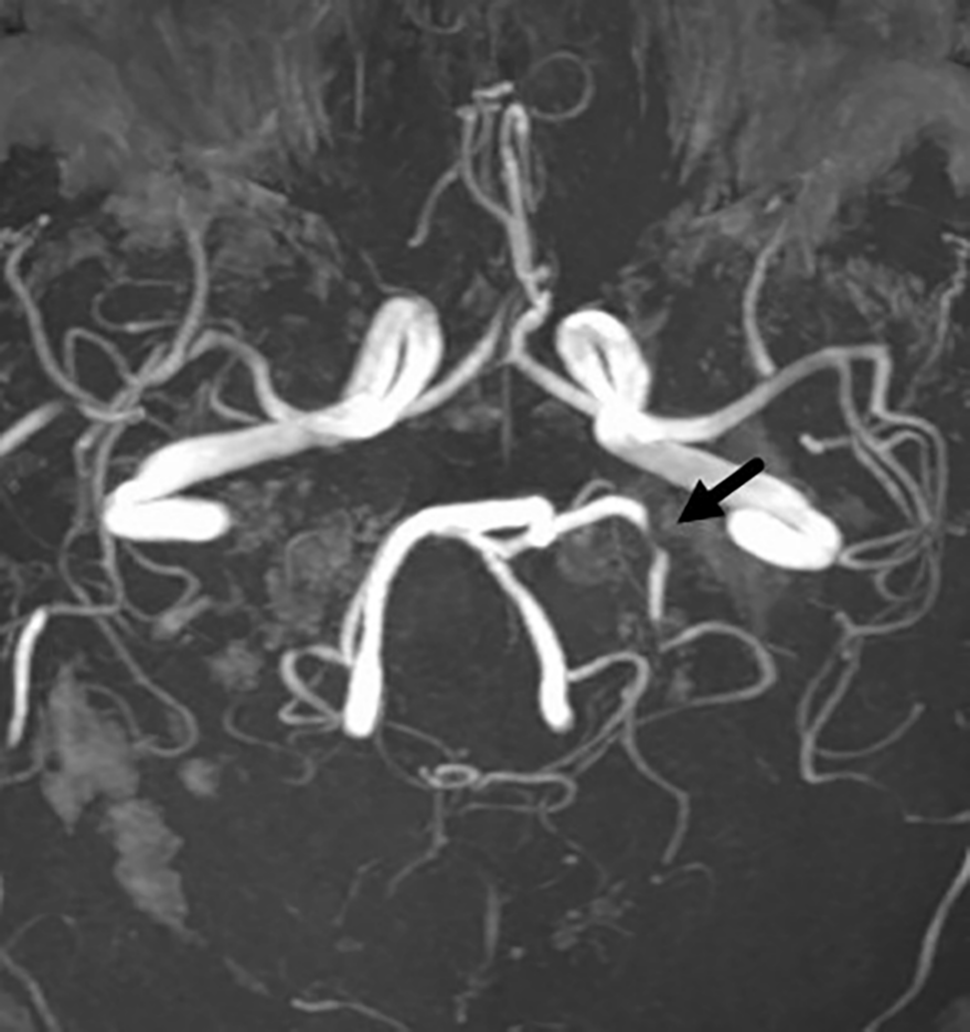
MIP TOF with an example of a left PCA stenosis

Atherosclerosis was seen in 39 patients. Eighteen of these represented significant stenoses, with luminal narrowing of 50–99% of pre-stenotic calibre (> 50%, *n* = 9, > 70% *n* = 9). The remainder (21) were non-stenotic cavernous carotid atherosclerosis. Of the 18 cases with significant stenoses, 12 had a CTA initially in additional to a non-contrast CT head. Intracranial atherosclerotic stenoses (ICAS) were noted on this initial imaging in 7 of these cases, but a new lesion was found at MRA in 5 patients (one case noted ICAS on index imaging; however, a significant lesion was found elsewhere on later MRA which had not been identified on index CT). All 5 cases were located in the posterior cerebral arteries (PCA), examples are given in Figs. 1 and 2. These PCA lesions were identifiable on the index CTA when the initial imaging was retroactively reviewed. Six patients with ICAS had no prior intracranial angiography (Table 2).

**Table 2:** Incidence of atherosclerosis causing > 50% luminal stenosis

ICAD	*N* = 18
MRA+ , CTA+	7
MRA+ , CTA −	5
MRA+ , no CTA	6

*CTA*+ atherosclerosis was seen on index CTA, *CTA*− atherosclerosis was not detected on index CTA

Intracranial aneurysms were seen incidentally in 14 (6.7%). Four of these patients had an initial CTA. Two of the aneurysms had been detected on the initial CTA, but in the other 2 cases, it was not seen on index imaging and was picked up at the time of MRA. The remaining 10 had no prior angiographic imaging before the MRA (Table 3).

**Table 3:** Rate of intracranial aneurysm detection

Intracranial aneurysms	*N* = 14
MRA+ , CTA+	2
MRA+ , CTA −	2
MRA+ , no CTA	10

*CTA* + aneurysm was seen on index CTA, *CTA* aneurysm was not detected on index CTA

## Discussion

Stroke is the second leading cause of death and the third leading cause of disability-adjusted life years lost worldwide [3]. Transient ischaemic attack is a recognised risk factor for stroke, with risk of stroke in the first 7 days post-TIA reported between 2.1 and 8% [4–6]. Early specialist assessment and treatment are associated with a reduction in this risk of up to 80% [3].

The presence of ICAD has been associated with an increased risk of recurrent stroke and disability following minor stroke and TIA [7–9]. Antiplatelet therapy, risk factor modification and lifestyle changes are the current standard of care for symptomatic ICAD [10]. Identification of a vascular culprit is crucial to inform management decisions and symptomatic patients remain at increased risk of recurrent events [11], although in patients with asymptomatic ICAD the risk of recurrence on standard medical therapy is comparable to TIA patients in whom no ICAD is identified [12]. In this study, the overall rate of significant ICAD was 8.6% (*n* = 18); ICAD was identified as a new finding at follow-up in 5.3%. The prevalence of ICAD in this group is somewhat lower than the prevalence seen in larger cohorts, for example in the OXVASC group, where the prevalence was 17.6% or 241 of 1368 patients [8]. The rate of additional ICAD detection on MRA may be amplified in these higher prevalence settings.

In patients who have not had any interrogation of their intracranial vasculature, the importance of MRA is clear; however, almost half of these cases had undergone initial CT angiography. The rate of additional ICAD detection at time of MR was 41% in those who had a CTA (5/12). Increased rates of ICAD detection balanced against the relatively short acquisition time of the TOF intracranial MRA (standard acquisition time in this institution was 4 min and 20 s) highlights the benefit of the follow-up TOF sequence. The inclusion of an MRA sequence in the MRI protocol for follow-up of suspected stroke and TIA provides a valuable opportunity to re-interrogate the intracranial vasculature.

Of note, all of the significant intracranial atherosclerotic stenoses that were identified at follow-up were located in the PCAs and were present on the index imaging when retroactively interrogated. A possible explanation for this inconsistency is that at the index presentation where CT is performed for suspected stroke and TIA, cases are read in an emergent setting or even on call. Often, inpatient teams are present requiring rapid assessment of the imaging in order for expedient decisions to be made regarding management. Radiological interpretation in this context may be prone to “type 1 thinking”, fast intuitive interpretation from experience [13]. This type of thinking is prone to bias, in this context perhaps satisfaction of search and inattention bias may be contributory which have been shown in one study of 1269 errors to account for 22% and 7% of imaging interpretation errors, respectively [14]. System-related errors such as interruptions, reading pace, distractions, visual and mental fatigue have been well documented in medicine and are undoubtedly a major contributory factor to radiological error and reduced diagnostic accuracy [13, 15]. These factors are likely to be amplified in the emergency setting. The follow-up MRA, on the other hand, takes place in outpatient setting, where timing is less critical, and the reading environment may lend itself to a calmer more analytical interpretation — “type 2 thinking” — perhaps accounting for some of the extra pick-ups. Regardless, the majority of errors in radiology are due to underreading or “misses”, 42% in the aforementioned study [14]. The use of checklists is thought to help alleviate this risk. This study serves as a reminder to ensure posterior cerebral arterial stenosis is on the TIA/minor stroke intracranial angiogram checklist.

There are several inherent weaknesses in this study. Firstly, this is a retrospective study and is prone to bias. Furthermore, this was undertaken in a single centre with a relatively small sample size which may be under-representative of the total population. A study involving multiple sites and readers would help to determine whether these findings are generalizable. In addition, this study examines the findings on TOF MRA and CTA, which is applicable to everyday practice; however, the availability of a gold standard reference technique such as digital subtraction angiography would be preferable for confirmation of the presence of ICAD.

Overall, the results of this retrospective assessment of over 100 patients who had undergone both CTA and MRA suggest that the MRA sequence, in providing an opportunity for a second look for an important stroke risk factor, conveys additional benefit to the outpatient MRI in those who have had CTA.

## Conclusion

The MRA component of the follow-up MRI in suspected TIA/stroke patients who have already had a CT angiogram of their intracranial vessels affords the radiologist a valuable opportunity for a second look at the intracranial vasculature. This results in increased detection rates for significant ICAD, an important risk factor which can affect management for TIA and stroke patients. The posterior cerebral arteries are an important check area for radiologists interpreting acute stroke.

## Data Availability

NA.

## Code availability

NA.
